# Particle on a Ring Model for Teaching the Origin of the Aromatic Stabilization Energy and the Hückel and Baird Rules

**DOI:** 10.1021/acs.jchemed.2c00523

**Published:** 2022-09-22

**Authors:** Miquel Solà, F. Matthias Bickelhaupt

**Affiliations:** † Institut de Química Computacional i Catàlisi and Departament de Química, Universitat de Girona, C/Maria Aurèlia Capmany, 69, 17003 Girona, Catalonia, Spain; ‡ Department of Theoretical Chemistry and Amsterdam Center for Multiscale Modeling, VU University, De Boelelaan 1083, 1081 HV, Amsterdam, The Netherlands; § Institute of Molecules and Materials (IMM), Radboud University, Heyendaalseweg 135, 6525 AJ Nijmegen, The Netherlands

**Keywords:** Introductory Chemistry, Physical Chemistry, Organic Chemistry, Quantum Chemistry, Chemical Concepts

## Abstract

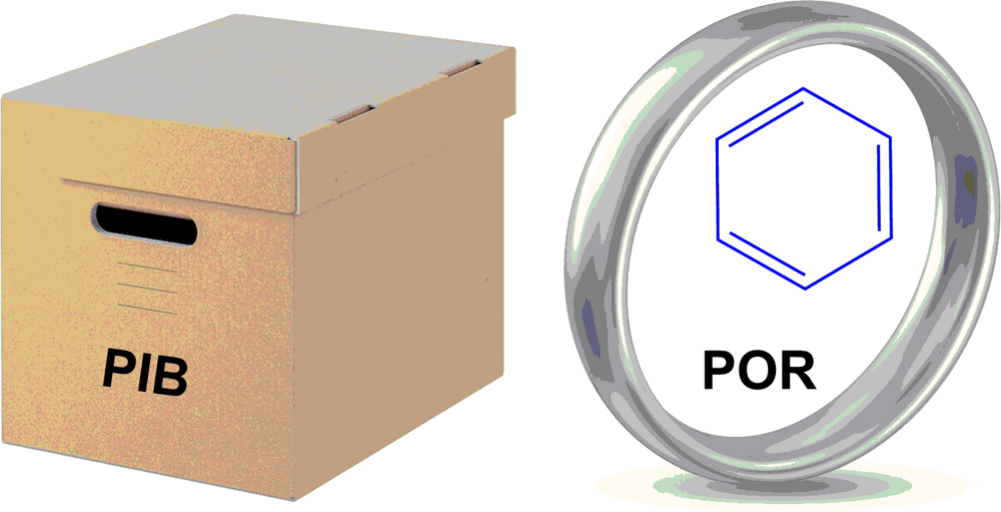

Simple mathematical models can serve to reveal the essence of experimental phenomena and scientific concepts. The particle in a box (PIB), for example, is widely used in undergraduate programs to teach the quantum mechanical principles behind the UV–vis spectra of conjugated polyenes and polyynes. In this work, the particle on a ring (POR) and the PIB models are used to elucidate the concept of aromaticity in Introductory Chemistry courses. Thus, we explain the origin of the aromatic stabilization energy, Hückel’s rule, and Baird’s rule. Besides applications, the limitations of the POR and PIB models are also discussed.

## Introduction

Arguably, the most important concept in chemistry is the chemical bond. For this reason, students learn about chemical bonding and theories that explain it relatively early in their academic career. The majority of basic chemistry courses begin the subject of chemical bonding with Lewis structures,^1^ which link atoms together through two-center two-electron (2c-2e) interactions, and with the valence-shell electron pair repulsion (VSEPR) theory,^2^ which gives students a sense of the three-dimensional shape of molecules based on the idea of minimizing the electron repulsion. These simple models, however, cannot account for the rich variety of chemical bonds. In particular, multicenter bonding in which three or more atoms share a pair of bonding electrons requires the introduction of Valence Bond (VB) theory or Molecular Orbital (MO) theory.^3^ Aromaticity, a type of multicenter bonding and a subset of chemical bonding theory, is usually described using either VB or MO theories or both.

Michael Faraday^4^ initiated the field of aromaticity in 1825, when he isolated benzene for the first time by distilling an oily residue. Since then, the concept of aromaticity has evolved and expanded from the ground states of stable compounds to transition states,^5^ from two dimensions to three dimensions,^6^ from the ground to excited states,^7,8^ from the main group elements to metals,^9^ and from the classical π-aromaticity to σ-, δ-, or even ϕ-aromaticity.^10^ The number of chemical species that can be classified as aromatic has increased enormously, especially in the past decades. Approximately two-thirds of all known chemical compounds are considered fully or partially aromatic.^11^ The growing interest in aromatic compounds and the concept of aromaticity, not only in organic chemistry but also in inorganic chemistry, is illustrated by the fact that, nowadays, more than 40 papers per day are published containing the word “aromatic*” in the title, abstract, or keywords.^12^

**Figure 1:**
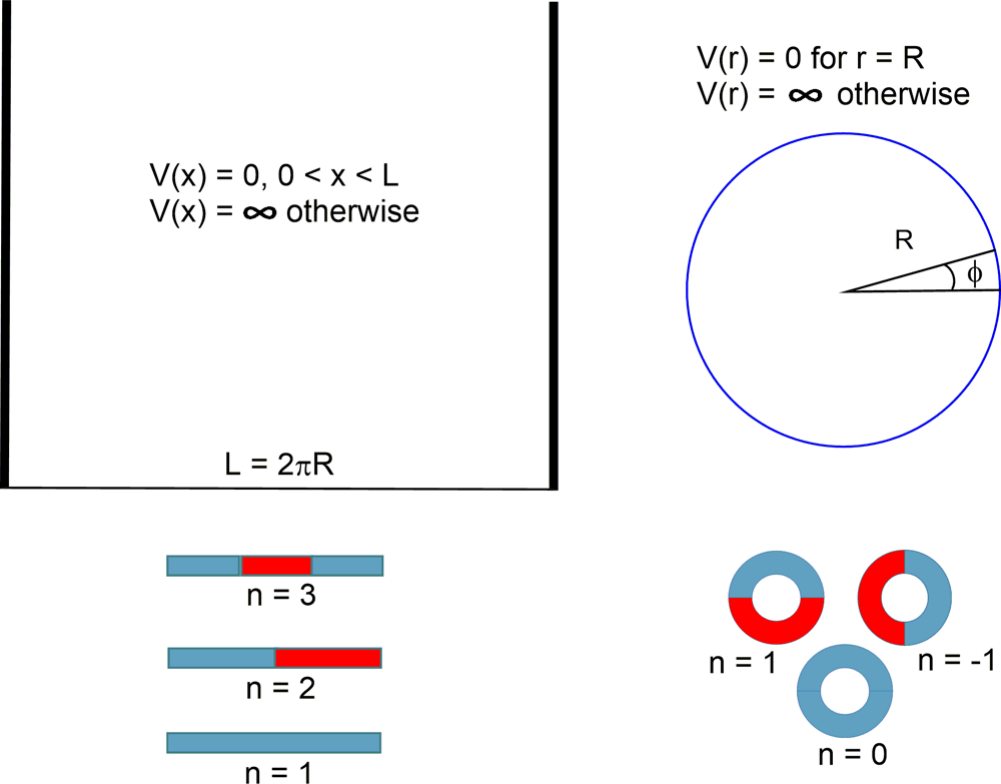
Particle in a 1-dimensional box (PIB, left panels) and particle on a ring (POR, right panels), with the corresponding potential wells (top panels) and the nodal structure of the first three most stable states (colors represent phase, bottom panels).

The aromaticity concept is a cornerstone to rationalize and understand the molecular structure, stability, and reactivity of a large number of compounds.^13^ Not surprisingly, therefore, this concept is presented already in Introductory Chemistry courses. Herein, we propose the particle in a 1-dimensional box (PIB) and the particle on a ring (POR) models^14^ as pedagogical principles for introducing and explaining this concept and the essential underlying quantum mechanics in an accessible manner to freshmen students (see Figure 1). Already in their first year, chemistry students learn how to solve the Schrödinger equation of the PIB model using pen and paper. Then, this PIB model is used in subsequent theoretical and practical courses to describe the UV–vis spectra of a number of linear π-conjugated compounds.^15^ Although the POR model is less known, solving the Schrödinger equation for the POR model represents essentially the same effort as solving the Schrödinger equation for the PIB model, if one performs a change from Cartesian to polar coordinates. The POR model can also be employed to describe quite accurately the UV–vis spectra of a number of monocyclic π-conjugated species.^16,17^

The PIB and POR models catch some, but not all, physics of the π-electron system of linear and monocyclic π-conjugated molecules, respectively. Although they are oversimplified models, they are able to reflect some of the major differences in the physical behavior of linear and monocyclic π-conjugated molecules. This makes the PIB and POR models useful tools for teaching the physical nature of aromaticity. The beauty of the PIB and POR models lies in their transparency which furnishes a didactical fashion of understanding the origin of the aromatic stabilization energy (ASE), Hückel’s rule, and Baird’s rule. Here, these concepts and rules are derived. And, importantly, the limits of the PIB and POR models are analyzed and discussed, as this is an integral part of the learning process.

## The Energy Levels of the Particle in a Box and the Particle on a Ring

For a particle of mass *m* in a 1-dimensional box of length *L*, the potential energy operator inside the box is zero, i.e., *V*(*x*) = 0 for 0 < *x* < *L*. This is the region along *x* where the particle can be. Outside the box, the potential is infinite, i.e., *V*(*x*) = ∞ for 0 ≥ *x* ≥ *L*, and the particle has zero probability in this region. This leads to a purely kinetic Hamiltonian for the PIB:^14^
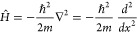
1associated with the boundary conditions that Ψ(0) = 0 and Ψ(*L*) = 0. The solution of the associated Schrödinger equation:
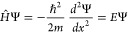
2is as follows:
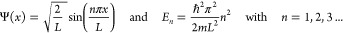
3The case *n* = 0 is to be excluded because in that case Ψ(*x*) = 0, which means that there is no particle in the box (see Figure 1, left). Note that negative *n* values lead to the same solution of the PIB problem as the corresponding positive *n* value; only the sign of the wave function is inverted.^14^

Next, let us consider the case of a particle of mass *m* on a ring of the same length, *L*, as the above 1-dimensional box, i.e., a ring with a radius *R* = *L*/2π. The potential energy operator on the ring is zero, i.e., *V*(*r*) = 0 for *r* = √(*x*^2^ + *y*^2^) = *R*. This is where the particle can be. Outside the ring, the potential is infinite, i.e., *V*(*r*) = ∞ for *r* ≠ *R*, and the particle has zero probability. This leads again to a purely kinetic Hamiltonian for the POR:^14,18^
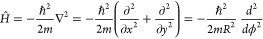
4where a change from Cartesian to polar coordinates (*x* = *R* cosϕ and *y* = *R* sinϕ) in the right-hand side of eq 4 has been introduced. This Hamiltonian is now associated with the boundary conditions that Ψ(*r* ≠ *R*) = 0. This Hamiltonian corresponds to that for a two-dimensional rigid rotor with mass *m* distributed on a ring of radius *R*.^14^ The Schrödinger equation that has to be solved for the POR is
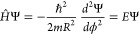
5associated with the periodic boundary condition Ψ(ϕ) = Ψ(ϕ + 2π) that must be satisfied to prevent self-extinction of the wave function. The solution of this Schrödinger equation is
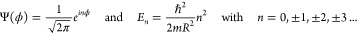
6If we take *L*′ = 2π*R*, then:^16^
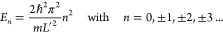
7and therefore for *L* = *L*′:
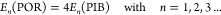
8

Note that, for the POR, *n* = 0 is a valid solution with an eigenfunction Ψ(ϕ) = (see below), with |Ψ(ϕ)|^2^ = 1/2π corresponding to a completely delocalized particle (the same complete delocalization is found for the rest of the levels). This is the quantum-mechanical analog of a particle standing still. This is at variance with the PIB for which the ground state has nonzero (kinetic) energy. This is the well-known ground-state energy and ground-state motion. A closely related phenomenon in chemistry is the ground-state, or zero-point, vibrational energy of the harmonic oscillator which differs from the PIB by having a parabolic potential box *V*(*x*) = *1/2kx*^2^ instead of a box with “hard walls” of *V*(*x*) = ∞ outside the box.

**Figure 2:**
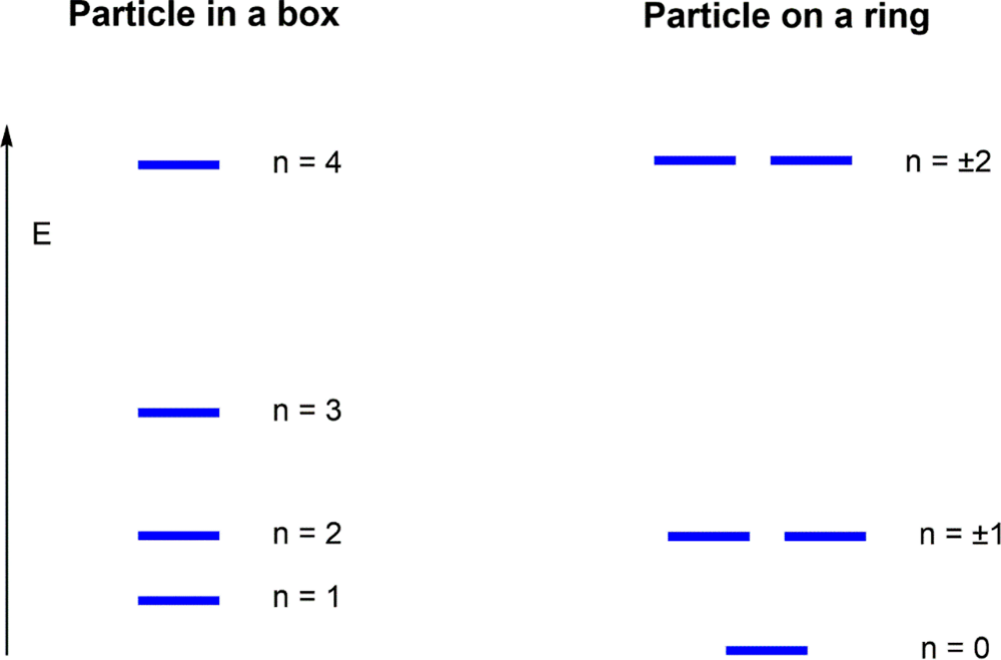
Energy levels for a particle in a 1-dimensional box and for the particle on a ring.

The energy levels for the PIB and POR are depicted in Figure 2. For the POR model, we use the linear combinations *e*^–*ik*ϕ^ + *e*^*ik*ϕ^ and *e*^–*ik*ϕ^ – *ie*^*ik*ϕ^ of the angular momentum eigenfunctions to represent the solutions of n = ±k as real functions. These linear combinations are still degenerate eigenfunctions of the Hamiltonian but not of the angular momentum operator. Note that this approach is analogous to the way in which the corresponding degenerate molecular orbitals of benzene are in general represented.^11^ There are two important differences that require our attention: (i) the just mentioned fact that the ground-state for the POR occurs for quantum number *n* = 0 (particle completely at rest), while this is not allowed for the PIB that has its ground-state for *n* = 1 (zero-point motion); and (ii) the POR has higher-energy solutions that come in pairs (*n* = ±1, ±2, ..., i.e., rotate left or right), whereas the PIB has no degenerate solutions (*n* = 1, 2, 3..., i.e., there are no different directions). The difference has to do with the fact that a particle on a ring with one particular energy can be in two distinct states associated with clockwise and counterclockwise rotation, whereas a particle in a 1-dimensional box cannot be in distinct states associated with motions in different directions; in classical terms, it moves left and right between the two ends of the box.

The energy levels obtained with the POR model can be used to calculate the promotion energy in benzene. For the promotion from *n* = 1 → 2, the POR model gives a wavelength of 207 nm, compared to an actual HOMO–LUMO UV absorption maximum for benzene of 204 nm.^16^

## Aromaticity: What Can Students Learn from the PIB and POR Models?

Once the energy levels for the PIB and POR have been derived, students can reach several conclusions that help their understanding of aromaticity. Let us consider a system with six particles in a box as an approximation to the 6 π-electrons of 1,3,5-hexatriene and six particles on a ring as an estimate of the 6 π-electrons of benzene. Taking the energy levels of Figure 2, the energy for the PIB with six particles is
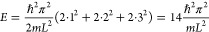
9If we consider *L*′ = 2π*R* for the circumference of the ring, the corresponding energy for the POR is
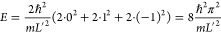
10The energy difference of the 6 π-electron POR versus PIB system can be taken, in a first approximation, as the ASE of benzene:
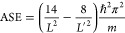
11This outcome shows us directly that, for *L* = *L*′, the POR model of an aromatic system is more stable than the corresponding PIB model of a linear conjugated species, with as the two foremost important reasons that a cyclic system has (i) a significantly more stable lowest-energy eigenvalue (i.e., particles at rest with zero kinetic energy) and (ii) degenerate higher-energy eigenvalues (i.e., they can accommodate twice as many particles per “shell” before the next higher-energy eigenfunctions must be populated). Thus, the POR and PIB model systems recover the existence of an ASE and provide insight into the underlying physics.

With POR and PIB models, the ASE of an *n*π-electron cyclic conjugated system can be estimated by evaluating Δ*E* of eq 11 using structural and mass data from experiment and the Aufbau principle of filling first the lowest energy levels, with a maximum of two particles per energy level.^19^ In the case of benzene, considering that the length of the box (linear π-conjugated system) and the circle (cyclic π-conjugated system) is the same, i.e., *L* = *L*′ in eq 11, and considering the perimeter of benzene as 6 times the experimental C–C bond distance in benzene, that is, 6 × 1.399 Å^20^ = 8.394 Å, and *m* as the mass on an electron, we get an ASE of 147.7 kcal/mol using eq 10. This value represents a clearly overestimated and rough approximation to the experimental ASE value obtained from different sources that ranges from 18.4 to 66.9 kcal/mol.^21^ The reason for the overestimation of the calculated ASE can be ascribed to the following approximations done: (i) the energy levels for POR and PIB models are strictly valid only for one particle and not for six interacting particles; (ii) the energy levels for POR and PIB models have been computed for particles on a ring, i.e., not in 3D spaces; and (iii) they do not consider the presence of nuclei nor the electron–electron interactions that reduce the delocalization and, consequently, the ASE. On the other hand, if one considers the length of the linear 1,3,5-hexatriene (6.958 Å)^22^ for the PIB instead of that of benzene (8.394 Å, i.e., one partially double C–C bond longer) in eq 10, the ASE is increased to 304.6 kcal/mol.

**Figure 3:**
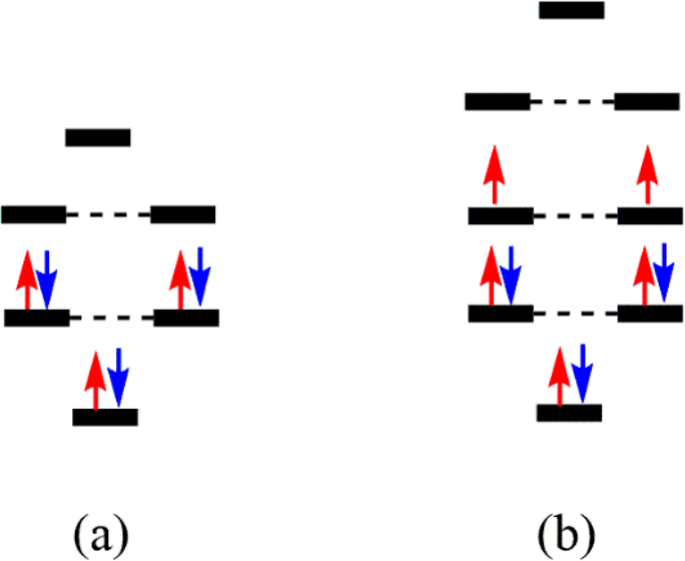
(a) Closed-shell six-electron POR model representing the π-system of benzene and (b) open-shell eight-electron POR model representing the π-system in the T_1_ state of the cyclooctatetraene.

Most of the typical aromatic compounds have high symmetry and degenerate molecular orbitals that can be fully occupied, resulting in a closed-shell structure. Or they can have the highest-energy occupied shell only half-filled with same-spin electrons. These two electronic distributions provide extra energetic stability that is the basis of aromaticity and the associated rules. With the molecular orbital distribution of the POR model, closed-shell electronic structures are obtained with 2, 6, 10, 14... π-electrons, i.e., with 4*N* + 2 π-electrons (*N* = 1, 2, 3...), resulting in the Hückel rule (see Figure 3a).^23^ Moreover, a last shell half-filled with same-spin electrons is reached with 4*N* π-electrons (see Figure 3b). According to the Baird rule,^7^ monocyclic D_nh_ annulenes with 4*N* π-electrons are aromatic in the lowest triplet state (T_1_). These rules can already be drawn from the simple POR model.

## Limitations of the Model

Several limitations of the POR and PIB models that arise because of the drastic simplifications made can be put forward. The simple potentials *V*(*r*) or *V*(*x*) neglect electron–electron interactions of the particles that simulate the π-electrons as well as those with other electrons in the real molecules. More importantly, they do not even account for the presence of nuclei separated by bond distances. One can partially remedy this latter problem by imposing that the circumference of the ring (POR) or length of the box (PIB) must be equal to experimentally known data. But this still precludes the description of phenomena such as bond length alternation in butadiene or bond length equalization in benzene. Also, the POR model does not explain the behavior of antiaromatic molecules such as cyclobutadiene or cyclooctatetraene. Even for these molecules, one gets a positive ASE. Second, the ASE per π-electron for a two π-electrons system is , for a six π-electrons system is , and for a 10 π-electrons system is . According to this model, the ASE increases with the size of the π-conjugated species. However, we know that the opposite behavior is found, i.e., the ASE per π-electron decreases with the size of the π-conjugated macrocycle.^24^ It is worth noting that *L* will increase with the number of π-electrons, so the trend found will be partially corrected if one accounts for the change in *L* with the increase in the number of π-electrons. And third, the Glidewell–Lloyd rule states that the total population of π-electrons in conjugated polycyclic systems tends to form the smallest 4*n* + 2 groups and to avoid the formation of the smallest 4*n* groups.^25^ In this sense, it is preferred to have six π-electrons in a smaller ring (in C_6_H_6_ better than in C_8_H_8_^+2^). However, the POR model favors the opposite trend, namely, the larger the length, *L*, the smaller the kinetic energy, and therefore the more stable the system is.

Despite these limitations, the simple POR model compared with the PIB model already offers a simple and pedagogic explanation of the quantum origin of the ASE and of the Hückel and Baird rules.

## Applications for Physical Chemistry Students

The preceding models and calculations are suited for Introductory or General Chemistry classes in which core concepts of chemistry, chemical bonding, and chemical reactivity are introduced and discussed. They serve, in particular, a deeper as well as a more intuitive understanding of how the concept of aromaticity is related to the underlying physical principles. Therefore, the material discussed herein also serves well in Physical Chemistry and in Organic Chemistry classes involving topics of chemical bonding and reactivity, in particular when it comes to, for example, the stability of aromatic compounds or the kinetics of reactions involving aromatic transition states. Last but not least, our discussion of the nature of aromaticity based on quantum-mechanical toy systems fits perfectly into Quantum Chemistry and Modeling. Teaching the aromaticity idea using the PIB and POR models helps students quickly grasp the essence of this and related concepts, such as the aromatic stabilization energy (ASE), Hückel’s rule, and Baird’s rule. This empowers students to recognize this phenomenon also in completely different situations, and it provides a sense of a unified physical theory behind chemistry.
